# Advancing Genome-Resolved Metagenomics beyond the Shotgun

**DOI:** 10.1128/mSystems.00118-19

**Published:** 2019-05-14

**Authors:** Rex R. Malmstrom, Emiley A. Eloe-Fadrosh

**Affiliations:** a Department of Energy Joint Genome Institute, Walnut Creek, California, USA

**Keywords:** DNA-SIP, genome-resolved metagenomics, microbiome

## Abstract

Exploration of environmental microbiomes has shed light on the ecological and evolutionary principles at play in natural ecosystems and has been further accelerated through the reconstruction of population genomes to provide genome-centric context. Yet technical challenges with traditional shotgun metagenomics remain for computationally intense short-read assembly, strain heterogeneity within communities, and depth of coverage required for low-abundance microbes. In this Perspective, we highlight three main avenues for promising future developments, including coupling stable isotope probing and genome-resolved metagenomics, applying fluorescence-activated cell sorting approaches to target mini-metagenomes within a larger community, and utilizing single-molecule long-read and synthetic long-read technology to link mobile elements to host microbial cells. These developments on the horizon will undoubtedly advance genome-resolved metagenomic approaches and enable a better understanding of uncultivated microbes in their natural environments.

## Abstract (precis)

Exploration of environmental microbiomes has shed light on the ecological and evolutionary principles at play in natural ecosystems and has been further accelerated through the reconstruction of population genomes to provide genome-centric context. Yet technical challenges with traditional shotgun metagenomics remain for computationally intense short-read assembly, strain heterogeneity within communities, and depth of coverage required for low-abundance microbes.

## PERSPECTIVE

The collection of bacterial and archaeal isolates with complete or draft genome sequences has reached a staggering mark with over 182,000 records currently available within public databases (1). This genomic inventory has provided a foundational reference of microbial metabolic and functional potential from which laboratory experimentation can validate sequence-based predictions. However, the vast majority of microbes have thus far eluded cultivation, and insights into their genetic makeup are available only through cultivation-independent approaches such as shotgun metagenomics and single-cell genomics. Recent advances in assembly and binning of metagenomic data sets at scale have enabled reconstruction of population-level draft genomes that provide significant insights into evolutionary and metabolic properties of uncultivated bacteria and archaea (2–5). Genome-resolved data analyses provide a unique opportunity to taxonomically anchor diverse microbiomes to enhance interpretation of community structure and function, and enable systems biology approaches and inform host-associated and Earth-system models.

One major limitation in applying genome-resolved metagenomic approaches lies with the untargeted, bulk nature of the initial sample collection. While we can now sequence deeper than ever before and assemble with increasingly powerful supercomputers, smarter approaches beyond bulk community shotgun sequencing are needed to gain access at the species and strain level and to associate functional capacity. We argue that more targeted, genome-resolved metagenomic approaches will move the field from hypothesis generation to a hypothesis-testing framework.

## COUPLING STABLE ISOTOPE PROBING AND GENOME-RESOLVED METAGENOMICS

DNA stable isotope probing (DNA-SIP) enables the targeted enrichment of active microbes based on uptake and incorporation of isotopically labeled (e.g., ^13^C, ^15^N, and ^18^O) substrates (6). In this approach, DNA extracted from communities incubated with isotopically labeled compounds is separated into different fractions along a cesium chloride density gradient such that higher-density fractions are enriched with “heavy” isotopically labeled DNA. Assimilation of labeled compounds can be inferred by changes in the density of a microbe’s DNA, thus making DNA-SIP a powerful tool for linking the identity of microbes to specific metabolic processes *in situ*.

Numerous DNA-SIP studies utilize high-throughput sequencing of 16S rRNA marker genes to explore links between phylogeny and *in situ* function (7–9). In these studies, 16S rRNA sequences are typically clustered into OTUs at 97% sequence similarity to mitigate methodological artifacts, yet these OTUs can be composed of distinct populations that differ substantially in gene content and activity. Shotgun sequencing of fractions enables genome-resolved DNA-SIP, where tracking labeled genomes instead of marker genes can distinguish functional activities among closely related, coexisting populations with high 16S rRNA similarity. More importantly, a genome-centric approach can provide insights into microbiome function that are not revealed using marker genes alone. That is, while 16S rRNA sequencing indicates who assimilated labeled substrates, a genome-centric approach also enables metabolic reconstructions that provide insights into how and why these substrates might be used in specific pathways.

In addition to functional insights, the fractionation steps associated with DNA-SIP can aid in overall genome recovery from complex communities. By nonrandomly dividing a community DNA into dozens of fractions before sequencing, it is possible to increase the relative abundance of some rare microbes in certain fractions, thus leading to greater coverage than would be found with shotgun sequencing of bulk DNA. For example, Starr and colleagues produced a closed genome of *Saccharibacteria* from a specific-density fraction, while this same genome had <1× coverage within the bulk metagenome (10).

Despite the potential power of DNA-SIP, its application has been somewhat limited, at least in part, by the laborious procedures involved. Development of automated DNA-SIP protocols would decrease variability while increasing throughput and overall accessibility. We predict advances in automation will stimulate a surge of studies measuring *in situ* functional activities of uncultivated microbial groups. In parallel, new informatic approaches are also needed to maximize assembly of genomes spread out over various fractions.

## TARGETED “MINI-METAGENOMES”

Complex microbial communities can also be divided into smaller, less diverse subsets before DNA extraction and sequencing. Microfluidic partitioning is a convenient method for generating mini-metagenomes by randomly separating small groups of cells into tiny reaction chambers prior to lysis and library creation (11). Fluorescence-activated cell sorting (FACS) is a more complicated but ultimately more flexible and precise method for randomly, or nonrandomly, generating mini-metagenomes. For example, using the latter approach, several genomes of uncultivated giant viruses were recovered from forest soils (12). Interestingly, these viral genomes could not be assembled by deep shotgun sequencing of the same soil samples, supporting the idea that subdivision of complex communities into low-diversity mini-metagenomes can enable recovery of rare members that might otherwise be overlooked using traditional bulk metagenomic approaches.

Regardless of cell separation method, the combination of mini-metagenomics and whole-community shotgun sequencing holds great promise for improving genome recovery from uncultivated microbial lineages. Incorporating contig coverage covariance among different samples dramatically improves metagenomic binning (13), but collecting the dozens of samples necessary to maximize power of coverage covariance can represent a substantial, and sometimes insurmountable, challenge. Subdividing a community into dozens of mini-metagenomes generates multiple samples with different phylogenetic composition, and algorithms could use coverage covariance among these mini-metagenomes to improve binning. For example, Yu and colleagues improved genome recovery by analyzing presence/absence patterns of cooccurring contigs found in several mini-metagenomes generated from a hot spring community (11). These cooccurrence and differential coverage patterns could also be used to bin contigs generated from corresponding whole-community shotgun sequencing. We believe there are great opportunities for improving genome recovery using a hybrid approach of mini-metagenomics and bulk metagenomics in a manner similar to the promising MetaSort method (14), while also better leveraging differential coverage patterns among mini-metagenomes.

Mini-metagenomics can also be combined with functional labeling to focus analyses on microbes of interest. Bio-orthogonal noncanonical amino acid tagging (BONCAT) is a method for fluorescently labeling cells that are actively synthesizing new proteins, and it can be coupled with FACS to generate mini-metagenomes composed solely of metabolically active microbes (15). Identifying and specifically sequencing active cells is critical for determining links between microbial genomes and environmental processes, especially in certain environments where at any particular time most cells may be growing very slowly, if at all. Raman-activated cell sorting (RACS) could also one day be used to specifically sort active cells, e.g., cells isotopically labeled during D_2_O incubations (16). In contrast to BONCAT+FACS, the combination SIP+RACS offers the possibility of generating mini-metagenomes based on more specific metabolic functions, e.g., identifying cells assimilating various ^13^C- or ^15^N-labeled organic compounds as opposed to simply “active” cells. Such an approach would be a powerful companion to DNA-SIP, in essence providing a digital complement to the analogue signal of DNA-SIP that produces greater clarity of cell-to-cell variability. SIP+RACS would also enable sorting and sequencing of cells assimilating compounds whose elements are not incorporated into nucleic acids, something not possible with DNA-SIP. Hopefully, engineering solutions enabling SIP+RACS on natural microbial communities will be realized in the future.

## LINKING MOBILE ELEMENTS TO MICROBIAL HOST CELLS

Beyond improved approaches for reconstructing genomes directly from the environment, new developments leveraging single-molecule long-read and synthetic long-read technology present unique opportunities to link mobile elements, specifically plasmids, to host microbial cells. Plasmid-mediated horizontal gene transfer impacts microbiome community structure and evolution, conveying distinct functional capabilities to microbes and exchanging genes among phylogenetic groups. Little is understood given the diversity of plasmids in terms of size, structure, and transmission mechanisms from natural populations. Limitations exist for direct isolation of plasmids from environmental samples, as well as accurate computational predictions using standard shotgun sequencing.

New tools hold great promise for establishing solid connections between microbial hosts and plasmids. For example, proximity-linking methods like Hi-C can physically connect plasmid DNA with host chromosomal DNA prior to library creation and sequencing, thus establishing a clear association between plasmids and host genomes (17). Microbial host-specific DNA methylation patterns can also identify plasmid sources. That is, different microbes often encode different methyltransferases that target different sequence motifs; thus, a plasmid’s host could be determined by matching methylation motifs. Beaulaurier and colleagues cleverly exploited this connection and used PacBio single-molecule, real-time (SMRT) sequencing to determine methylation motifs of plasmid and chromosome sequences from synthetic and natural microbiomes, and were able to link plasmids to hosts (18). While few studies have leveraged single-molecule long-read and synthetic long-read technology to link plasmids to their respective host microbial cells, we anticipate these approaches will gain traction in the future to expand our current knowledge of diverse plasmids.

Together, advances in targeted genome-resolved metagenomic approaches are increasingly providing ways to capture greater resolution within environmental microbiomes. Through genome-resolved DNA-SIP and mini-metagenomics approaches, functional activity measurements can be directly linked to uncultivated microbial groups in a genome-centric manner and bring us increasingly closer to *in situ* hypothesis testing. Similarly, alternative sequencing strategies using single-molecule long-read and synthetic long-read technology afford a means to target mobile genetic elements linked to hosts and further expand our knowledge of uncultivated microbes.
